# Idiopathic gigantomastia in a patient on polypharmacy

**DOI:** 10.1259/bjrcr.20210052

**Published:** 2021-06-09

**Authors:** Ngoc-Nhu Jennifer Nguyen, Lilia Maria Sanchez, Mariam Yassa, Julie David, Mona El Khoury

**Affiliations:** 1 Université de Montréal, Montreal, Quebec, Canada; 2 Department of Pathology, Centre Hospitalier de l’Université de Montréal, Montreal, Quebec, Canada; 3 Department of Surgical Oncology, Centre Hospitalier de l’Université de Montréal, Montréal, Quebec, Canada; 4 Department of Radiology, Centre Hospitalier de l’Université de Montréal, Montréal, Quebec, Canada

## Abstract

Gigantomastia is an uncommon benign condition characterized by massive breast enlargement. It is most often due to hormonal imbalance secondary to puberty or pregnancy, or induced by a pharmacological agent but can also be idiopathic. Herein, we report a rare case of idiopathic gigantomastia in a 46-year-old female on antiepileptic multiple-drug therapy who underwent total bilateral mastectomy to relieve associated pain.

## Case presentation

A 46-year-old premenopausal female presented with a 2-year history of progressive asymmetrical enlargement of the breasts, with the right breast being more markedly enlarged than the left one. She complained of back pain and right mastalgia for the past 6 months. She did not report any nipple discharge or palpable mass. She weighed 100 kg and was 170 cm tall, with a body mass index (BMI) of 34.6 kg/m^2^.

The patient was known for post-encephalitic intractable multifocal epilepsy for which she had been taking many medications since the age of 27. She had a vagus nerve stimulatat age 37 and a corpus callosotomy at age 38. Upon presentation, her antiepileptic therapy comprised of clobazam, levetiracetam, oxcarbazepine and lorazepam. She was also on solifenacin, acetaminophen, folic acid, aripiprazole, buproprion, venlafaxine, olanzapine, pantoprazole, alendronate, calcium, vitamin D and naproxen. She had one child of 10 years old whom she had not breastfed and a history of a spontaneous abortion. During her pregnancies, the patient had appropriate breast dimensions.

**Figure 1.**
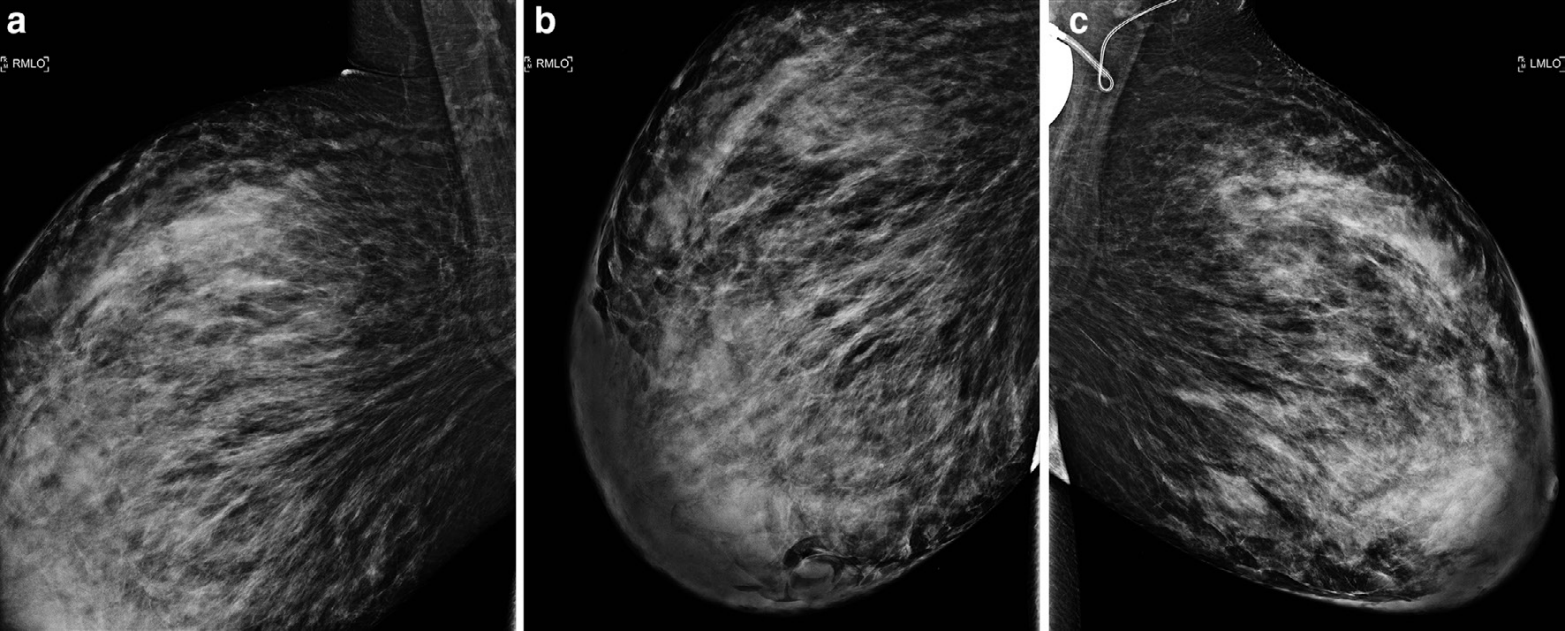
(**a-c**): Mediolateral oblique mammograms of the right (**a,b**) and left (**c**) breast obtained at the time of referral, demonstrating heterogeneously dense breast parenchyma with diffuse skin and trabecular thickening. Note the asymmetrical increase in volume of the breasts compared to the previous mammogram obtained two years prior as displayed in Figure 2. Also note the vagal nerve stimulator projecting over the left major pectoral muscle.

**Figure 2.**
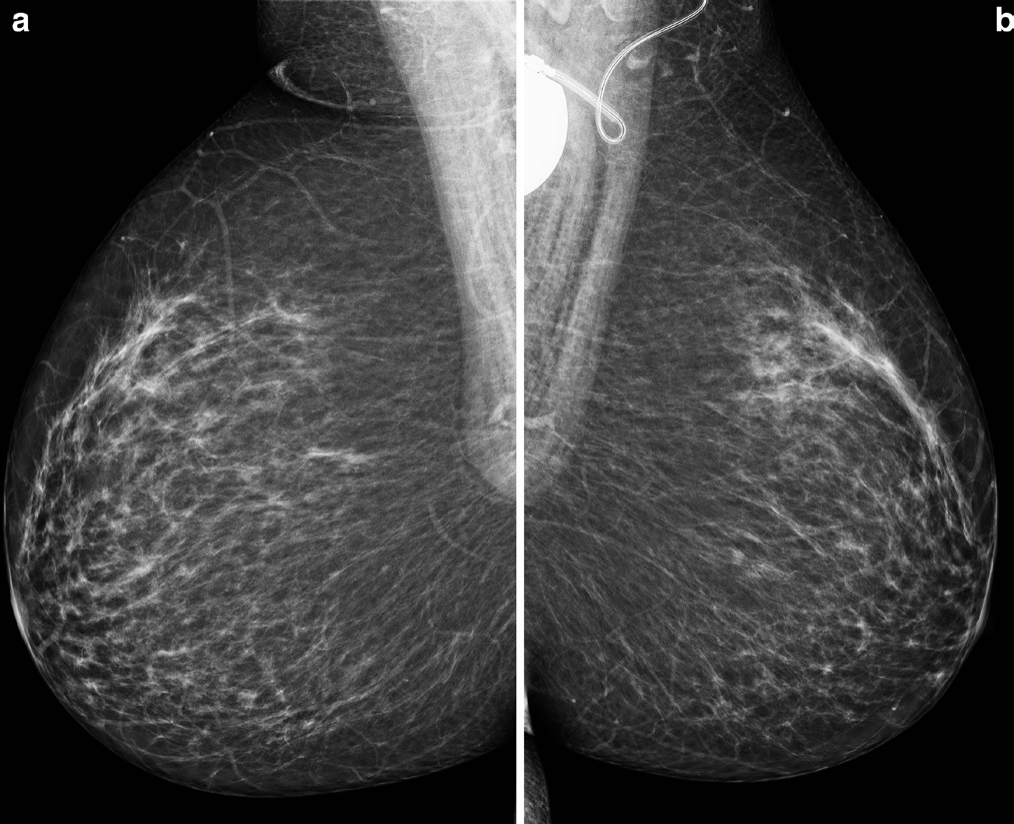
(**a,b**): Mediolateral oblique mammograms of the right (**a**) and left (**b**) breast obtained two years prior demonstrating symmetrical and homogeneous low-density breasts.

**Figure 3.**
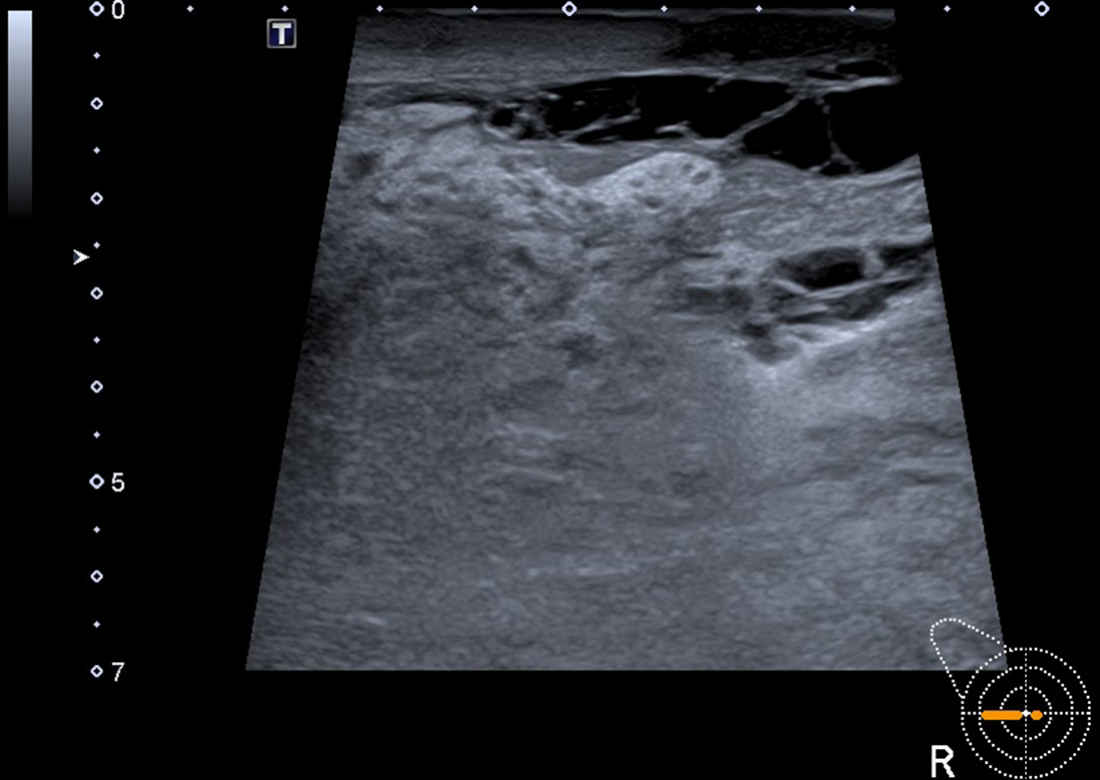
Grayscale ultrasound image of the right breast displaying skin thickening and anechoic subcutaneous structures with thin septations that could be dilated lymphatic ducts or cystic changes, but that were most likely veins on Doppler mode.

**Figure 4.**
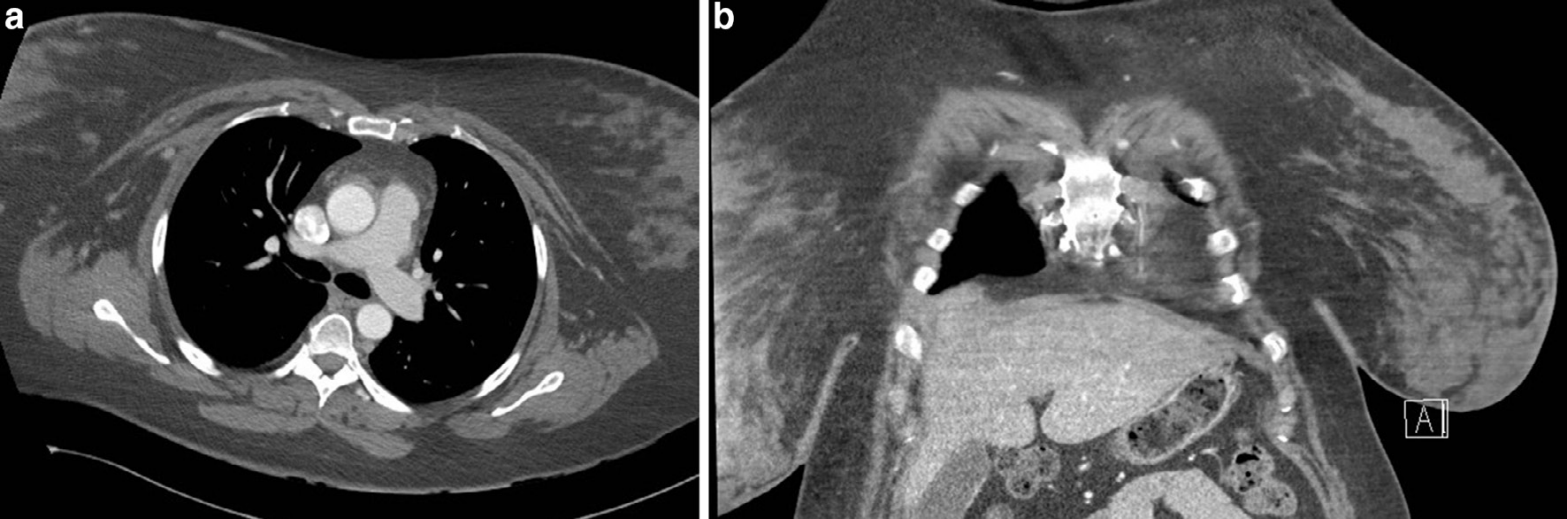
(**a,b**): Axial (**a**) and coronal (**b**) images of the thoracic CT angiogram demonstrating large breasts, more so on the right, partially included in the field of view.

On clinical examination, the breasts were enlarged, especially the right one, with a “peau d’orange” aspect due to bilateral diffuse cutaneous edematous infiltration. The skin was thick and pitted, especially the nipple-areolar complexes, with no colour change noted. On mammography, both breasts have significantly increased in volume (Figure 1) in comparison with the previous bilateral mammogram performed two years priorly (Figure 2). With this increase in size, bilateral, progressive diffuse densities that were more confluent in the retroareolar regions and anterior third of the breasts, have appeared. Moreover, there was now a cutaneous thickening predominating in the anterior third of the breasts associated with diffuse trabecular thickening in keeping with an edematous infiltration. Sonographic evaluation was limited due to the large breasts’ volume and the edematous skin infiltration but shows multiple hypoechoic nodular areas associated with bilateral, anechoic, serpentine subcutaneous structures that proved to be venous on Doppler ultrasound (Figure 3). No axillary masses or adenopathies were seen. Central venous obstruction or thrombosis was suspected but ruled out by a CT angiogram of the thorax that showed again the significant breast enlargement (Figure 4). Malignancy, especially ovarian cancer, which is known to rarely metastasize to the breast as an inflammatory breast cancer,^1,2^ was excluded by an abdominal CT scan. A breast MRI could not be performed for further evaluation because the patient had a vagal nerve stimulator.

Laboratory studies were within normal limits

Considering the pain and discomfort associated with the progressive breast enlargement and the newly developed mastitis of the right breast, the patient was referred to plastic surgery. She was offered a bilateral reduction mammoplasty and informed of the risk of recurrence. After reflection, she requested a total bilateral mastectomy with nipple areolar complex sparing that was performed two months after her last imaging studies.

**Figure 5.**
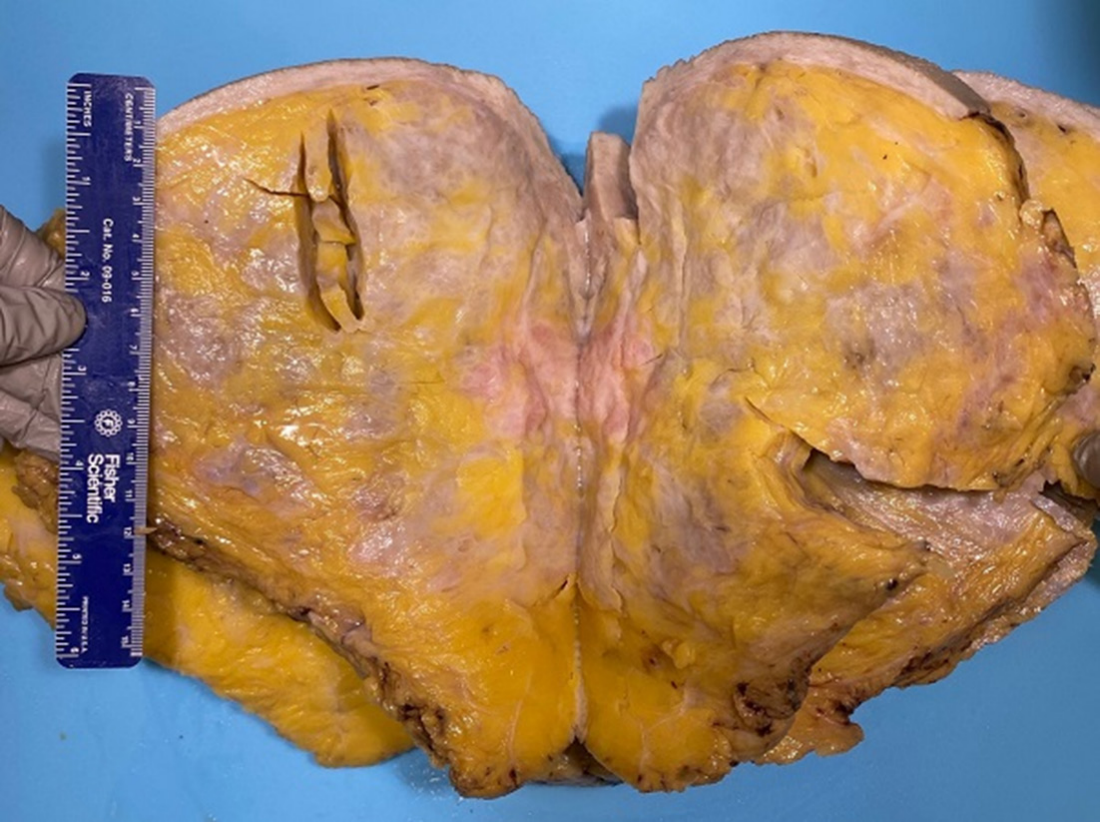
Cut surfaces of the right breast specimen displaying dense white fibrous tissue and yellowish adipose tissue.

**Figure 6.**
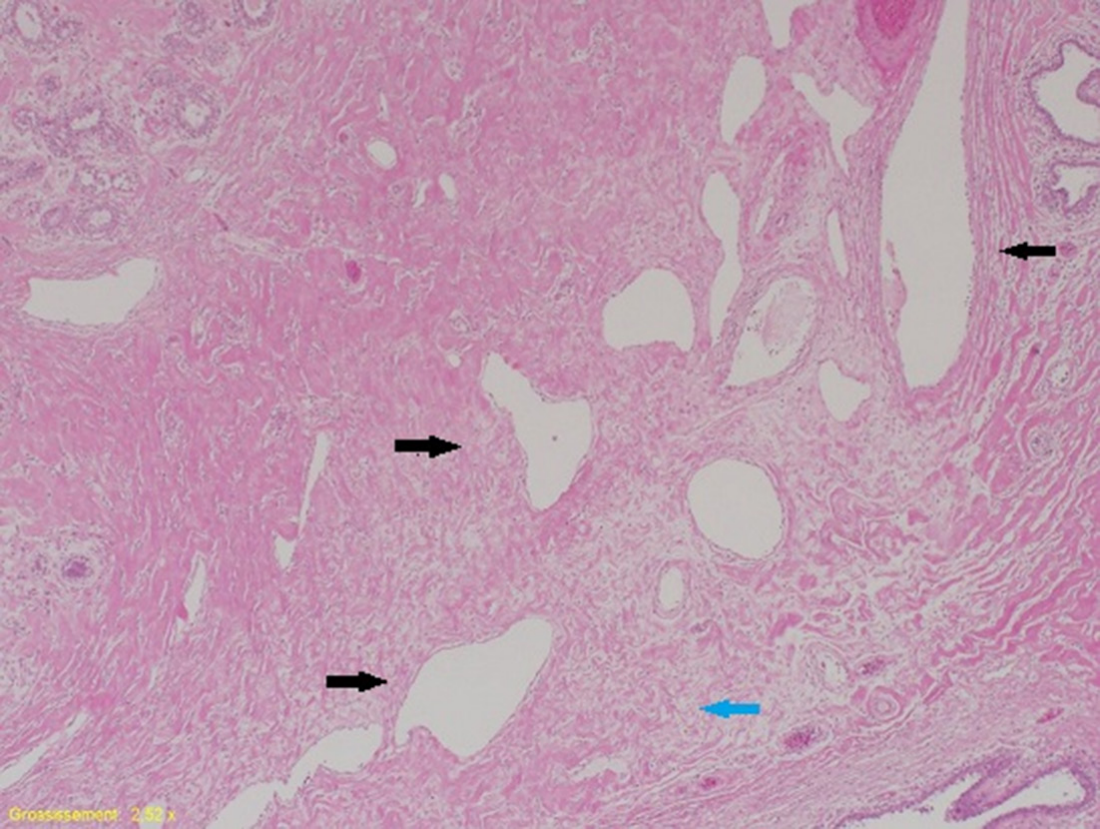
Histological examination of the right breast demonstrating diffuse edematous infiltration (blue arrow) with dilated lymphatics in the glandular parenchyma (black arrows) (H&E, original magnifications ×2 [A] and ×4 [B]).

The specimen from the right side weighed 4250 g and measured 220 × 200 × 170 mm. The specimen from the left side weighed 2690 g and measured 210 × 200 × 150 mm. The epidermal surface was unremarkable. On section, the breast parenchyma of both breasts was composed of about 80% of dense white fibrous tissue and 20% of a yellowish adipose tissue that contained around 20 cystic or microcystic formations measuring 0.5 cm at the most (Figure 5). Histological examination revealed apocrine metaplasia associated with a pseudoangiomatous stromal hyperplasia (PASH) pattern that was patchy, not tumorous, representing around 20 and 15% of the right and left breast tissues, respectively. The dominant finding was, however, dilated lymphatic vessels in the dermis, subcutaneous tissue and glandular parenchyma with diffuse edematous infiltration (Figure 6). No atypia or carcinoma cells were seen.

## Differential diagnosis

The association of trabecular and skin thickening with breast enlargement suggest an inflammatory involvement of the breasts. As such, the differential diagnosis includes an inflammatory breast cancer which is usually unilateral and associated with erythema and axillary lymphadenopathies.^3,4^ Secondary inflammatory involvement of the breasts from an extramammary primary such as ovarian or lung cancer through lymphatic spread was ruled out by CT scan.^1,2^

Edematous infiltration secondary to central venous obstruction or thrombosis was considered in the differential diagnosis and was ruled out by angio CT scan of the thorax.

## Discussion

Gigantomastia is a rare disabling benign condition characterized by massive breast enlargement.^5^ It was first described in 1960 by Lewison et al. but up until now, there is still no universal consensus for its definition.^5^ Many authors define it as an excessive breast growth of more than 1.5 kg, but the threshold ranges between 0.8 and 2 kg depending on the author.^5^ According to Dafydd et al., gigantomastia is defined as an excess breast tissue representing at least 3% of the body weight.^6^

Dancey et al. in 2008 classified gigantomastia into three main types, whereby Type one is idiopathic, related to massive breast enlargement in females with a BMI over 30 (Type 1a) or less than 30 (Type 1b), Type two is due to hormonal imbalance secondary to puberty (Type 2a) or pregnancy (Type 2b), and Type three is induced by a pharmacological agent.^5^

Gigantomastia can also be seen in association with autoimmune diseases, including rheumatoid arthritis, Hashimoto thyroiditis, myasthenia gravis and psoriasis.^5,7^

Despite our patient’s multiple medications, none of her drugs was incriminated for gigantomastia^5,8^ and she was not known for any systemic inflammatory disease. Thus, her breast enlargement was most likely idiopathic of Type 1b. Das et al. suggested that idiopathic gigantomastia is attributable to paracrine effects of estrogen given normal hormonal levels demonstrated by quantitative immunofluorescence in the three cases of idiopathic gigantomastia they reported and its occurrence in a postmenopausal female.^9^

In our patient, histological analysis shows the presence of PASH, representing around 20 and 15% of the breast tissue of the right and left breast respectively. It was, however, not tumorous, nor diffuse to account for the breast enlargement. PASH is usually an asymptomatic, incidental microscopic finding, identified in up to 23% of breast specimens.^10^ It sometimes presents as a dominant palpable mass that resembles a fibroadenoma or phyllodes tumor, which is then referred as tumorous or nodular PASH.^11^ Rarely, it presents as diffuse bilateral breast enlargement without any nodular mass, known as diffuse PASH.^11,12^ Diffuse tumorous PASH, which demonstrates features of both, is an even much rarer presentation and could be associated with gigantomastia.^11^

The diffuse interstitial and peri-ductal edematous infiltration encountered in our patient initially raised the hypothesis of either central venous obstruction such as the superior vena cava syndrome or the infrequent secondary inflammatory neoplastic involvement of the breasts from an extramammary primary malignancy such as ovarian cancer,^1,2^ but they were both ruled out by CT scan. This diffuse interstitial and peri-ductal edematous infiltration, together with lymphatic and terminal duct dilatation, were also described in idiopathic gigantomastia,^9,13^ as well as in juvenile and pregnancy-induced gigantomastia.^14,15^

As in our patient, gigantomastia can lead to mastalgia, back pain, postural difficulties and psychological problems but also skin ulceration and infection as well as loss of nipple sensation due to chronic traction of the fourth, fifth and sixth costal nerves.^5,7,10^

The first-line treatment is usually breast reduction but breast enlargement often reoccurs, most likely in overweighed patients with a BMI over 30.^5^ A mastectomy as a definitive treatment might then be needed.^5^ Surgical excision is sometimes combined with hormonal therapy.^5^ Bromocriptine and tamoxifen have been shown to cause regression of the breast size, in gestational pregnancy and juvenile gigantomastia, respectively.^5^ It has also been reported that medroxyprogesterone and dydrogesterone are successful in slowing breast enlargement.^5^ Hormonal therapy success rates remain however variable.^5^

## Conclusion

Gigantomastia is a rare multifactorial clinical condition characterized by massive enlargement of the breasts to an extent that despite the fact it is benign, a total mastectomy is sometimes needed for symptomatic relief.

## Learning objectives

1. Gigantomastia is a rare disabling entity characterized by excessive breast overgrowth of more than 1.5 kg or at least 3% of the body weight.
2. Gigantomastia is either idiopathic (Type 1), related to hormonal imbalance secondary to puberty (Type 2a) or pregnancy (Type 2b), or secondary to medications (Type 3).
3. Although hormonal therapy can be effective as in puberty or pregnancy-related gigantomastia, surgery may sometimes be required.
